# A comparative effectiveness trial of postoperative management for lumbar spine surgery: changing behavior through physical therapy (CBPT) study protocol

**DOI:** 10.1186/1471-2474-15-325

**Published:** 2014-10-01

**Authors:** Kristin R Archer, Rogelio A Coronado, Christine M Haug, Susan W Vanston, Clinton J Devin, Christopher J Fonnesbeck, Oran S Aaronson, Joseph S Cheng, Richard L Skolasky, Lee H Riley, Stephen T Wegener

**Affiliations:** Department of Orthopaedic Surgery and Rehabilitation, Vanderbilt University Medical Center, Nashville, TN USA; Department of Biostatistics, Vanderbilt University Medical Center, Nashville, TN USA; Department of Neurological Surgery, Vanderbilt University Medical Center, Nashville, TN USA; Department of Orthopaedic Surgery, Johns Hopkins Medicine, Baltimore, MD USA; Department of Physical Medicine and Rehabilitation, Johns Hopkins Medicine, Baltimore, MD USA; Department of Orthopaedic Surgery and Rehabilitation, Vanderbilt University, School of Medicine, Medical Center East – South Tower, Suite 4200, Nashville, TN 37232 USA

**Keywords:** Comparative effectiveness research, Cognitive-behavioral therapy, Self-management, Rehabilitation, Postoperative pain, Spine surgery

## Abstract

**Background:**

The United States has the highest rate of lumbar spine surgery in the world, with rates increasing over 200% since 1990. Medicare spends over $1 billion annually on lumbar spine surgery. Despite surgical advances, up to 40% of patients report chronic pain and disability following surgery. Our work has demonstrated that fear of movement is a risk factor for increased pain and disability and decreased physical function in patients following lumbar spine surgery for degenerative conditions. Cognitive-behavioral therapy and self-management treatments have the potential to address psychosocial risk factors and improve outcomes after spine surgery, but are unavailable or insufficiently adapted for postoperative care. Our research team developed a cognitive-behavioral based self-management approach to postoperative rehabilitation (*Changing Behavior through Physical Therapy (CBPT))*. Pilot testing of the CBPT program demonstrated greater improvement in pain, disability, physical and mental health, and physical performance compared to education. The current study compares which of two treatments provided by telephone – a CBPT Program or an Education Program about postoperative recovery - are more effective for improving patient-centered outcomes in adults following lumbar spine surgery for degenerative conditions.

**Methods/design:**

A multi-center, comparative effectiveness trial will be conducted. Two hundred and sixty patients undergoing lumbar spine surgery for degenerative conditions will be recruited from two medical centers and community surgical practices. Participants will be randomly assigned to CBPT or Education at 6 weeks following surgery. Treatments consist of six weekly telephone sessions with a trained physical therapist. The primary outcome will be disability and secondary outcomes include pain, general health, and physical activity. Outcomes will be assessed preoperatively and at 6 weeks, 6 months and 12 months after surgery by an assessor masked to group allocation.

**Discussion:**

Effective rehabilitation treatments that can guide clinicians in their recommendations, and patients in their actions will have the potential to effect change in current clinical practice.

**Trial registration:**

NCT02184143.

**Electronic supplementary material:**

The online version of this article (doi:10.1186/1471-2474-15-325) contains supplementary material, which is available to authorized users.

## Background

The United States (U.S.) has the highest rate of lumbar spine surgery in the world, with rates increasing over 200% since 1990 among adults over age 60 years with degenerative spinal disease [1–3]. Medicare spends over $1 billion annually on lumbar spine surgery and fusion procedures account for almost half [4]. Despite surgical advances, adults undergoing lumbar spine surgery have poorer physical and mental health outcomes compared to the general population [5, 6]. More specifically, up to 40% report persistent pain, functional disability and poor quality of life and 20% to 24% undergo a reoperation [7–10].

Providers routinely offer physical therapy after spine surgery, without high-quality evidence that this postoperative approach improves outcomes [11]. Several randomized trials have found no significant difference between standard physical rehabilitation and either no treatment or an educational booklet [12–14]. These trial results may be due to the inability of physical therapy to address the psychosocial factors often associated with poor surgical spine outcomes. Our work and that of others has demonstrated that fear of movement is a risk factor for increased pain and disability and decreased physical function in patients following lumbar spine surgery for degenerative conditions [15–18]. Fear of movement refers to an excessive fear of physical activity resulting from a dysfunctional belief that movement will cause harm or reinjury [19].

Cognitive-behavioral therapy interventions have strong empirical support, with positive influence on fear of movement, as well as pain catastrophizing and self-efficacy in chronic pain populations [20, 21]. Studies have demonstrated that brief and telephone-administered cognitive-behavioral programs are effective for reducing pain and improving function in patients with chronic and surgical pain [22–26]. Cognitive-behavioral based self-management programs have also demonstrated improvement in patient outcomes and the adoption of a physically active lifestyle, as well as improvement in fear-avoidance beliefs and self-efficacy in various populations with chronic conditions [27, 28].

To date, cognitive-behavioral and self-management treatments have not been tested in a surgical spine population. Two studies have investigated alternative approaches to traditional physical therapy in patients following surgery for lumbar degenerative conditions. Christensen et al. [29] studied a “Café Group” intervention (i.e., peer support) and found significantly lower leg pain but not back pain at 2-year follow-up compared to a video group and 8 weeks of exercise training. Abbott et al. [30] found decreased disability with a 3-session psychomotor therapy program (i.e., motor relearning) compared to a home program at 2 years. However, the psychomotor program did not demonstrate a significant effect on pain and health-related quality of life outcomes [30]. Both studies suggest that intensive and supervised exercise programs are not needed to improve outcomes after spine surgery. Furthermore, the North American Spine Society (NASS) suggests that there is insufficient evidence to support the use of active physical therapy or exercise as a stand-alone treatment strategy [18].

Preliminary work from our lab indicates the potential for a sizeable benefit of a cognitive-behavioral based self-management approach to postoperative rehabilitation relative to current practice in patients following lumbar spine surgery for degenerative conditions [31]. Our program – *Changing Behavior through Physical Therapy (CBPT*) - is designed to engage patients in their own care, improve shared postoperative decision-making, and maximize gains in outcomes that are relevant and meaningful to patients. Pilot testing of our CBPT Program demonstrated greater improvement in pain, disability, physical and mental health, and physical performance compared to education.

The purpose of this study is to compare which of two treatments provided by telephone – *CBPT Program* focusing on self-management or an *Education Program* about postoperative recovery - are more effective for improving patient-centered outcomes in adults following lumbar spine surgery for degenerative conditions. The primary aims of this study are to: 1) compare the effectiveness of a CBPT Program and an Education Program for improving pain, disability, general health, and physical activity, 2) determine how the CBPT Program improves outcomes and 3) determine which subgroups of patients are most likely to benefit from the CBPT Program.

## Methods/design

### Funding

This study has received funding from the Patient-Centered Outcomes Research Institute (PCORI) (CER-1306-01970).

### Study design

**Figure 1:**
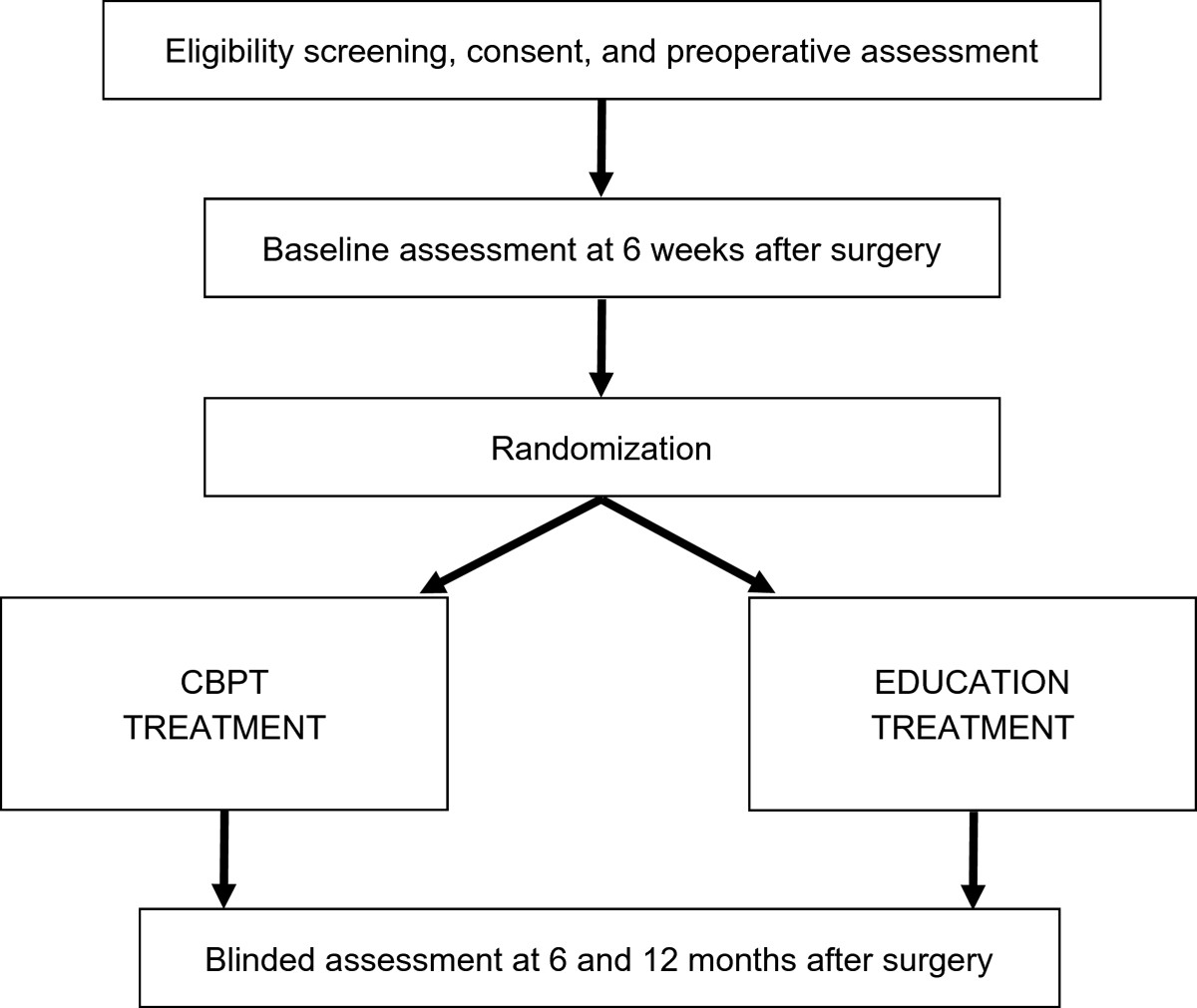
**Flow diagram of the comparative effectiveness trial.**

This multi-center, prospective randomized controlled trial has primary recruitment sites in Nashville, TN and Baltimore, MD. Figure 1 illustrates the overall study design with assessments preoperatively and at 6 weeks (baseline), 6 months and 12 months after lumbar spine surgery (see ClinicalTrials.gov and NCT02184143 for more information).

### Ethical principles

Ethical approval has been received from the Institutional Review Boards of Vanderbilt University and Johns Hopkins Medicine. Written informed consent will be obtained from all participants prior to study enrollment.

### Study population

Two hundred and sixty English-speaking adults who are having surgical treatment of a lumbar degenerative condition (spinal stenosis, spondylosis with or without myelopathy, and degenerative spondylolisthesis) using laminectomy with or without arthrodesis (i.e., fusion) procedures will be recruited for this study. Participants will be recruited from clinical sites at two academic medical centers (Vanderbilt University Medical Center and Johns Hopkins Medicine). Additional recruitment will occur from community orthopaedic surgery practices located in Tennessee and Kentucky.

### Exclusion criteria

Patients will be excluded from the study if they meet any of the following criteria:

-  A microsurgical technique as the primary procedure, such as an isolated laminotomy or microdiscectomy.
-  Spinal deformity as the primary indication for surgery.
-  Spine surgery secondary to pseudarthrosis, trauma, infection, or tumor.
-  Back and/or lower extremity pain < 3 months indicating no history of sub-acute or chronic pain.
-  History of neurological disorder or disease, resulting in moderate to severe movement dysfunction.
-  Presence of schizophrenia or other psychotic disorder.
-  Surgery under a workman’s compensation claim.
-  Not able to return to clinic for standard follow-up visits with surgeon.
-  Unable to provide a stable address and access to a telephone.

### Randomization

A computer-generated scheme will randomize patients in a 1:1 ratio in blocks of assignments frequency matched on age and type of surgery (i.e., fusion or no fusion), resulting in 4 strata: (1) Age 21-59 and fusion; (2) Age 60-90 and fusion; (3) Age 21-59 and no fusion; (4) Age 60-90 and no fusion. Block size will be determined randomly with the patient as the unit of randomization. Randomization will be administered centrally by Vanderbilt through the Research Electronic Data Capture (REDCap) system [32]. Randomization will occur immediately following the baseline assessment at 6 weeks after surgery. Participants along with the research coordinators, surgeons, and other research personnel responsible for data collection will be unaware of randomization assignment.

### Comparators

#### CBPT treatment

The CBPT Program focuses on a patient-oriented self-management approach to reduce pain and disability and improve physical activity, through reductions in fear of movement and increases in self-efficacy (Table 1). Brief cognitive-behavioral programs for pain developed by Woods and Asmundson [33], Williams and McCracken [34], and Turner et al. [35] and a self-management approach developed for older adults by Lorig [36, 37] provide the basis for the CBPT treatment. Sessions cover an introduction and rationale for treatment, deep breathing [38], progressive muscle relaxation [39], graded activity plan, goal-setting [40], distraction techniques [34], automatic thoughts [41], coping self-statements [41], pacing techniques [42], and relapse prevention and symptom management plans [43]. Each session builds upon the content of the previous session and weekly action plans are personally tailored based on patient goals. The CBPT Program consists of six weekly telephone sessions with a trained physical therapist. The first session is 60 minutes and the remaining 5 sessions are 30 minutes. Each patient randomized to the CBPT Program will receive a binder to follow along with the study therapist (see http://www.spine-surgery-recovery.com for more information).

**Table 1:** **Summary of the CBPT treatment by session**

*Topics*	*Major content and activities*
All Sessions include: Graded Activity; Goal Setting; Problem-Solving	Each session builds upon the content of the previous session. Format includes: 1) review of previous session personally tailored activity and walking goals and skills homework, 2) problem-solving barriers to completing goals, 3) introduction of new content through discussion and worksheets, and 4) review of homework assignment to be completed before next session.
**Session 1:** **Goal Setting**	Review purpose of the program, conduct semi-structured patient interview, complete a graded activity plan and fear hierarchy, set activity goals based on hierarchy, explore walking history and set walking goals, introduce deep breathing as pain management strategy.
Introduction; Establish a Graded Activity Plan and Fear Hierarchy; Deep Breathing	
**Session 2:** **Your Mind and Recovery**	Check graded activity plan, review activity and walking progress and set new goals, problem-solve barriers to completing goals, introduce distraction as pain management strategy and complete worksheet, introduce progressive muscle relaxation CD.
Distraction Techniques; Progressive Muscle Relaxation	
**Session 3:** **Balance your Thinking**	Review activity and walking progress and set new goals, problem-solve barriers to completing goals, introduce event-thoughts-feeling-action handout, identify negative thoughts that effect activity using worksheet, practice replacing negative thoughts with positive self-talk and complete worksheet.
Identify Negative Thoughts; Positive Self-Statements	
**Session 4:** **Rest and Activity**	Review activity and walking progress and set new goals, problem-solve barriers to completing goals, review activity types handouts, explore pacing strategies for pain management and complete worksheet, identify benefits of program so far and complete worksheet.
Activity Types; Pacing; Benefits of Program	
**Session 5:** **Managing Setbacks**	Review activity and walking progress and set new goals, problem-solve barriers to completing goals, review relapse cycle handout, complete managing setbacks worksheet.
Relapse Prevention Plan	
**Session 6:** **Staying Healthy**	Review activity and walking progress, problem-solve barriers to completing goals, complete pain management plan worksheet, reinforce importance of regular exercise and follow-up visits with surgeon and other health care providers.
Pain Management Plan; Wrap-up	

#### Education treatment

The Education Program focuses on postoperative recovery and consists of modules that were developed and tested in a National Institutes of Health (NIH) funded trial for patients with musculoskeletal injury (R01AR054009). Educational modules were adapted in collaboration with adults who completed preliminary testing of the CBPT treatment [31]. Sessions address benefits of physical therapy, proper biomechanics after surgery, importance of daily exercise, and ways to promote healing. Education on stress reduction, sleep hygiene, energy management, communication with health providers, and preventing future injury are also provided (Table 2). The education treatment is matched to the CBPT treatment in terms of session frequency, length and contact with the study physical therapist. Each patient randomized to the Education Program will receive a binder to follow along with the study therapist (see http://www.spine-surgery-recovery.com for more information).

**Table 2:** **Summary of the education treatment by session**

*Topics*	*Major content and activities*
**Session 1:** **Physical Therapy**	Review purpose of the program, conduct semi-structured patient interview, describe physical therapy, introduce benefits of physical therapy, describe different physical therapy techniques, and introduce different exercise programs.
**Session 2:** **Promote Back Healing I**	Discuss importance of proper posture and transitions, describe proper sleeping positions, and introduce ways to promote healing.
**Session 3:** **Promote Back Healing II**	Discuss importance of proper body mechanics, describe proper lifting techniques, and describe proper ergonomics at home and at work.
**Session 4:** **Home Exercise Program**	Describe the importance of a home exercise program (HEP), discuss the goals of a HEP, introduce the components of a HEP, and discuss the benefits of a HEP.
**Session 5:** **Prevent Future Injury**	Discuss ways to prevent reinjury, describe mechanisms of low back strain, and introduce ways to manage a low back strain.
**Session 6:** **Staying Healthy**	Describe ways to stay healthy, discuss specific benefits of exercise and not smoking, and discuss ways to reduce stress, improve sleep, eat healthier, and conserve energy.

#### Quality assurance

One study physical therapist (SWV) at Vanderbilt will complete a formal training course in both the CBPT and educational treatments. Formal training will occur during one 2-day session with the PI of the study (KRA) and a clinical psychologist (STW) specializing in cognitive-behavioral and self-management techniques. A written competency for both treatments and a skills test for the CBPT Program will be completed at the end of training. Both treatments will be implemented with study staff and progress will be discussed during weekly research meetings. A formal pre-test of the CBPT Program and Education Program will then occur with 2 patients. All sessions during the pre-test will be audiotaped and reviewed by the PI (KRA) and a clinical psychologist (STW) to evaluate adherence to the treatment protocol and specific CBT competencies [44].

Our treatment integrity protocol includes: 1) therapist training and competence in delivering the treatments and in the importance of fidelity; 2) use of detailed treatment manuals; and 3) ongoing supervision to ensure accurate and consistent treatment delivery (provided via weekly clinical team meetings). The study physical therapist’s adherence to procedures will be assessed by audio-recording all sessions and randomly selecting sessions (balanced evenly across the sessions) for the investigators to review and rate treatment integrity using a standardized fidelity checklist. The PI (KRA) and a clinical psychologist (STW) will oversee the ratings and checklist to provide corrective feedback to the study therapist as needed in real time. The study therapist (SWV) will also complete a checklist of all the components delivered during each session and make note of any protocol deviations. If the integrity of the treatments is compromised, the study therapist will be re-trained and 100% of audiotapes will be reviewed until problems are addressed.

#### Data collection

Table 3 summarizes key data collection across time points. Self-report assessments will be conducted before surgery and at 6 weeks, 6 months, and 12 months after surgery. Questionnaires will be completed in clinic or remotely using a REDCap survey. Movement accelerometers will be used to measure physical activity at the 6 week, 6 month, and 12 month time-points. Accelerometers will be provided to patients in clinic at 6 weeks after surgery and through the mail at 6 and 12-month follow-up.

**Table 3:** **Data collection schedule**

		Postoperative		
	Preoperative	6 Week	6 Month	12 Month
**Patient Characteristics**				
Age, Gender	X			
Race/Ethnicity	X			
Marital Status	X			
Educational Level	X			
Insurance Status	X			
Height/Weight	X			
Smoking Status	X			
Working status	X	X	X	X
**Medical History**				
Pain Duration	X			
Prior Spinal Surgery	X			
Comorbidities	X			
Current medications	X	X	X	X
**Surgical Characteristics**				
Type (fusion/no fusion)	X			
Spinal Levels	X			
Revision Surgery	X			
**Psychosocial**				
Fear of Movement	X	X	X	X
Pain Self-Efficacy	X	X	X	X
Depressive Symptoms	X	X	X	X
Expectations	X			
Satisfaction			X	X
**Outcomes**				
Disability	X	X	X	X
Pain	X	X	X	X
General Health	X	X	X	X
Physical Activity		X	X	X
**Health Services**				
Physical Therapy		X	X	X
Re-hospitalization		X	X	X
Additional Surgery		X	X	X

### Primary outcome measure

#### Disability

The 10-item Oswestry Disability Index (ODI) is a standard measure of condition-specific disability that assesses the impact of lumbar spinal disorders on various aspects of daily life [45]. Ratings for each item are from 0 (high functioning) to 5 (low functioning). Total scores are divided by the total possible score and multiplied by 100 to create a percentage of disability. The ODI has demonstrated strong test-retest reliability and validity, and good internal consistency in both surgical spine patients and patients with chronic low back pain [46, 47]. The minimum clinically important difference (MCID) has been found to range from 11 to 12.8 points in patients following lumbar spine surgery [48, 49].

### Secondary outcomes measures

#### Pain intensity and interference

The Brief Pain Inventory (BPI) will assess pain intensity and pain interference with activity [50]. The 4-item pain intensity subscale assesses current, worst, least, and average pain. For this trial, only the average pain item will be used to assess pain intensity. A single-item rating of average pain has been found to be as valid for detecting treatment effects as a variety of composite scores (i.e., current, worst, least and average), especially for large trials that involve group comparisons [51, 52]. The 7-item pain interference subscale assesses general activity, mood, walking ability, normal work, relations with other people, sleep, and enjoyment of life. Both subscales use a numerical rating scale with 0 representing ‘no pain or does not interfere’ and 10 representing ‘pain as bad as you can imagine or completely interferes’. Scores greater than or equal to 5 indicate moderate to severe pain intensity and interference. The BPI has proven both reliable and valid in both surgical patients and patients with chronic low back pain [53–55]. The MCID for pain has been found to range from 1.2 to 2.1 points in patients following lumbar spine surgery [48].

#### General health

General physical and mental health will be measured with the physical and mental composite scales of the SF-12 [56]. The physical component scale (PCS) assesses the four subdomains of physical functioning, role-physical, bodily pain, and general health and the mental component scale (MCS) assesses the 4 subdomains of vitality, social functioning, role-emotional, and mental health. Total subscale scores range from 0 to 100, with 100 indicating the highest level of health. The PCS and MCS of the SF-12 have demonstrated responsiveness, good test–retest reliability, good internal consistency, and validity in generalized and various patient populations [56–58]. The minimal clinically significant change for the PCS and MCS has been estimated at 10% [58].

#### Physical activity

Physical activity will be measured objectively using a commercially available movement accelerometer (ActiGraph GT3X-BT) [59]. Accelerometers are used in physical activity monitoring because of their small size, low cost, convenience, the ability to record data for several days [60], and ability to assess multiple dimensions of physical activity [61, 62]. Accelerometers have proven valid with moderate correlations with the criterion method of doubly labeled water for total and active energy expenditure in young and older adults [63, 64]. Physical activity will be assessed using total volume of physical activity, expressed as the mean counts per minute over the duration of accelerometer monitoring. In addition, percentage of time spent in commonly used domains of physical activity intensity (sedentary, light, moderate, and vigorous) will be considered.

### Additional measures

#### Fear of movement

A shortened version of the Tampa Scale for Kinesiophobia (TSK) will be used to measure fear of movement [65]. For this trial, the 4 reversed scored items (4, 8,12, 16) were omitted. Psychometric research supports the removal of these items to improve internal consistency, factor structure, and goodness of fit of the TSK [66]. A total score can range from 13 to 52. Participants are asked to rate each item on a 4-point Likert scale with scoring alternatives ranging from ‘strongly disagree’ to ‘strongly agree’. The MCID for the TSK has been reported to be 4 points in patients with back pain [67]. The TSK has been found to have good internal consistency and test-retest reliability in surgical patients and patients with various musculoskeletal conditions [68, 69].

#### Pain self-efficacy

The Pain Self-Efficacy Questionnaire (PSEQ) will measure the strength and generality of a person’s belief in his/her ability to accomplish a range of activities despite pain [70]. Participants rate how confident they are on a 7-point scale from ‘not at all confident’ to ‘completely confident’. Scores range from 0 to 60, with a score greater than 40 indicating high pain self-efficacy [71]. The PSEQ has been found to have excellent internal consistency, good test-retest reliability, and construct validity through correlations with depression, anxiety, coping strategies, pain ratings, and work-related tasks in patients with chronic pain [70].

#### Depressive symptoms

The 9-item Patient Health Questionnaire-9 (PHQ-9) will be used to assess signs and symptoms that are characteristic of major depression [72]. The total score can range from 0 to 27 with higher numbers indicating higher depressive symptoms. Participants rate each item on a 4-point Likert scale with scoring alternative ranging from ‘not at all’ to ‘nearly every day’. The PHQ-9 has excellent reliability and is a sensitive and specific measure of major depression in primary care [72, 73].

### Data analysis

#### Comparative effectiveness of treatments

Data will be explored statistically and graphically. Group means and corresponding confidence intervals will be calculated for baseline variables, to confirm balance between groups. The characteristics of the patients who are lost to follow-up will be compared to those who complete the follow-up assessments. For each outcome variable, we will fit a longitudinal mixed-effects model, with a random intercept for patient to account for the correlation among observations from the same patient and a group random effect to account for variation among centers. We will explore possible non-linear (i.e., quadratic, cubic) effects of the treatment over time. A random slope over time may be included to allow a separate slope to be estimated for each patient. We will fit the model with an independent conditional covariance structure and an autoregressive structure and choose the best data-supported model based on the deviance information criteria or a related criterion. The primary analysis will be intent-to-treat; missing observations due to dropout and other reasons not related to the treatments will be handled with multiple imputation methodology.

#### Potential mediators

Separate longitudinal mixed-effects models will be used to explore associations between changes in fear of movement and pain self-efficacy from baseline to 6 months and changes in outcomes from baseline to 12 months after surgery for the entire sample. We will construct mediation models that estimate the effect of the mediation by changes in fear of movement and in pain self-efficacy on outcomes as the result of surgery. This comprises 3 sub-models that relate (1) the treatment to health outcomes directly, (2) the treatment to fear of movement and pain self-efficacy and (3) both the treatment and fear of movement and pain self-efficacy to health outcomes simultaneously. This will demonstrate any mediation effect, if present, by seeing how the relationship between treatment and outcomes change when the potential mediators are added or removed.

#### Subgroup effects

Longitudinal mixed-effects models will be used to explore the interaction between patient characteristics and treatment for each outcome in the entire sample. Important subgroups will be identified based on the strength of association between the response to treatment (change in outcomes) and each covariate included in the model (i.e., patient age, type of surgery, depressive symptoms).

### Sample size

We estimated power for all aims of the study, based on a target of 110 patients per arm with complete follow-up data at 12 months. Power was estimated by generating simulated data, then using the simulated data to try to estimate the original model parameters. We generated 200 simulated datasets by resampling available pilot data from a NIH funded project (R21AR062880). Control subjects were resampled from control individuals in the pilot data, and treatment subjects were also resampled from control individuals, but with the target effect size added to the sampled values. Power was estimated by fitting Bayesian models to each of the simulated datasets for each response variable and recording the proportion of calculated 95% credible intervals for effect sizes that excluded zero. There will be sufficient power to detect the following effect sizes: 7.0 points on the 0-100 ODI, 1.5 points on the 0-10 BPI, 30% for the accelerometer, 4.0 points on the 13-52 TSK, 6.0 points on the 0-60 PSEQ, and 10% main effect for subgroup covariates. To account for a 15% patient drop out rate, a total of 260 subjects will be enrolled into the study.

## Discussion

Adults undergoing lumbar spine surgery for degenerative conditions continue to have poor outcomes following surgery, with up to 40% reporting residual chronic pain and disability. Psychosocial factors, in particular fear of movement, have been found to be significant risk factors for poor long-term outcomes. Cognitive-behavioral and self-management treatments show promise in reducing psychosocial risk factors, but are unavailable or insufficiently adapted for postoperative care. Currently, there are no evidence-based programs that clinicians can recommend and patients can do after spine surgery to improve outcomes. There is an urgent need for accessible treatments that empower patients to take an active role in their care and reduce psychosocial risk factors in order to prevent long-term disability and chronicity after spine surgery. Effective rehabilitation treatments that can guide clinicians in their recommendations, and patients in their actions, will have the potential to effect change in current clinical practice.

The aim of this comparative effectiveness study is to conduct a rigorous evaluation of a physical therapist delivered cognitive-behavioral based self-management program with the goal of engaging adults in their own care and improving pain, disability, general health and physical activity outcomes. A randomized controlled trial design will be used to address the central hypothesis that a CBPT Program focusing on self-management will improve surgical spine outcomes, through reductions in fear of movement and increases in self-efficacy (i.e., belief in ability to perform certain behaviors). Results will further our understanding of tailored physical therapy treatments for health outcomes.

This study will be the first to investigate a cognitive-behavioral based self-management approach to postoperative spine rehabilitation. Our proposed study is timely, because the physical therapy treatment paradigm is shifting away from pain relief to pain management through “psychologically informed” rehabilitation and compelling data are needed to support this expanded scope of practice [74, 75]. Our interventional approach seeks to redefine the transdisciplinary model of health care and broaden the availability of effective pain management and behavior change strategies by expanding the implementation from traditional providers (i.e., psychologists) to physical therapists. The long-term goal is to increase access to evidence-based, patient-centered treatments that maximize outcomes in the postoperative setting.

## Authors’ information

KRA, PhD, DPT is Assistant Professor of Orthopaedic Surgery and Physical Medicine and Rehabilitation and Director of Orthopaedic Research at Vanderbilt University Medical Center.

RAC, PT, PhD is a Research Fellow in the Department of Orthopaedic Surgery at Vanderbilt University Medical Center.

CMH is a Clinical/Translational Research Coordinator in the Department of Orthopaedic Surgery at Vanderbilt University Medical Center.

SWV, PT, MS is a Research Physical Therapist in the Department of Orthopaedic Surgery at Vanderbilt University Medical Center.

CJF, PhD is Assistant Professor of Biostatistics at Vanderbilt University Medical Center.

CJD, MD is Assistant Professor of Orthopaedic Surgery and Director of the Spinal Column Surgical Quality and Outcomes Research Laboratory at Vanderbilt University Medical Center.

OSA, MD, MMHC is Associate Professor and Residency Program Director of Neurological Surgery and Medical Director of the Vanderbilt Spine Center at Vanderbilt University Medical Center.

JSC, MD, MS is Associate Professor of Neurological Surgery and Director of Neurosurgery Spine Program at Vanderbilt University Medical Center.

RLS, ScD is Associate Professor of Orthopaedic Surgery and Director of Spine Outcomes Center at Johns Hopkins Medicine.

LHR, MD is Professor of Orthopaedic Surgery, Chief of Orthopaedic Spine Division and Vice-Director for Clinical Orthopaedic Surgery Operations at Johns Hopkins Medicine.

STW, PhD is Associate Professor of Physical Medicine and Rehabilitation and Director of Division of Psychology and Neuropsychology at Johns Hopkins Department of Physical Medicine and Rehabilitation.

## Authors’ original submitted files for images

Below are the links to the authors’ original submitted files for images. Authors’ original file for figure 1

## Abbreviations

U.S.: United States
NASS: North American Spine Society
CBPT: Changing Behavior through Physical Therapy
PCORI: Patient-Centered Outcomes Institute
REDCap: Research Electronic Data Capture
NIH: National Institutes of Health
ODI: Oswestry Disability Index
BPI: Brief Pain Inventory
PCS: Physical Component Scale
MCS: Mental Component Scale
TSK: Tampa Scale for Kinesiophobia
PSEQ: Pain Self-Efficacy Scale
PHQ-9: 9-item Patient Health Questionnaire.
