# Supplementary material for: A comparative effectiveness trial of postoperative management for lumbar spine surgery: changing behavior through physical therapy (CBPT) study protocol
Source: BMC Musculoskelet Disord. 2014 Oct 1;15:325. doi: 10.1186/1471-2474-15-325 (PMC4192328; doi:10.1186/1471-2474-15-325)
Supplement: Supplementary file 1 — Authors’ original file for figure 1 [file 12891_2014_2261_MOESM1_ESM.pdf]

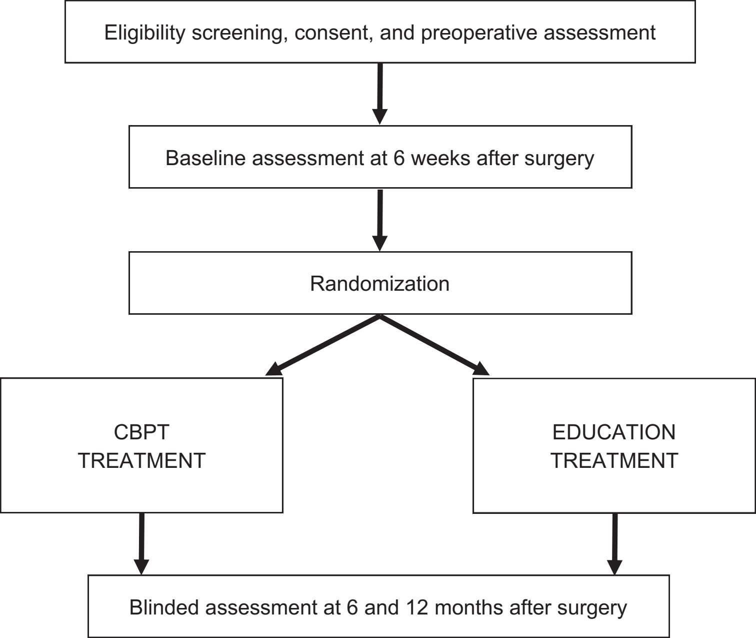

Eligibility screening, consent, and preoperative assessment
Baseline assessment at 6 weeks after surgery
Randomization
CBPT TREATMENT
EDUCATION TREATMENT
Blinded assessment at 6 and 12 months after surgery
